# A high-efficiency Mg-1Bi-0.5Nd anode magnesium alloy for discharge applications

**DOI:** 10.1038/s41598-025-33101-8

**Published:** 2025-12-26

**Authors:** Zheng Jia, Tingting Song, Xiaowei Niu, Fu Yang, Sichao Du

**Affiliations:** 1 College of Mechanical Engineering, Shenyang University, Shenyang, 110044 China; 2 College of Environment, Liaoning Province Pollution Environmental Remediation Professional Technology Innovation Center & Shenyang Key Laboratory of Collaborative Technology Innovation for Industrial Pollution Reduction and Carbon Reduction, Shenyang University, Shenyang, 110044 China

**Keywords:** Rare earth elements, Magnesium-air battery, Electrochemical performance, Discharge performance, Chemistry, Energy science and technology, Materials science

## Abstract

A novel homogeneous Mg-1Bi-0.5X (X = Gd, Y, Nd, Ce, La) alloy was fabricated in this study, and its electrochemical properties as well as discharge characteristics as an anode material for primary magnesium-air batteries were systematically investigated through electrochemical tests and battery discharge experiments. The results demonstrate that the addition of Nd promotes the formation of a refined grain structure and semi-continuous network-distributed NdBi secondary phase, which significantly enhances the discharge performance. Microstructural analysis reveals that the discharge products of Mg-1Bi-0.5Nd exhibit a loose, porous morphology. This structure facilitates product exfoliation via crack propagation, thereby improving electrolyte accessibility and enhancing the anode’s discharge stability and efficiency. At a current density of 20 mA cm^−2^, the alloy achieves a specific capacity of 1492.54 mAh g^−1^ and an anodic efficiency of 68.5%, representing a 114.1% improvement over that of the AZ31. These results highlight the superior discharge performance of the Mg-1Bi-0.5Nd anode.

## Introduction

**Fig. 1:**
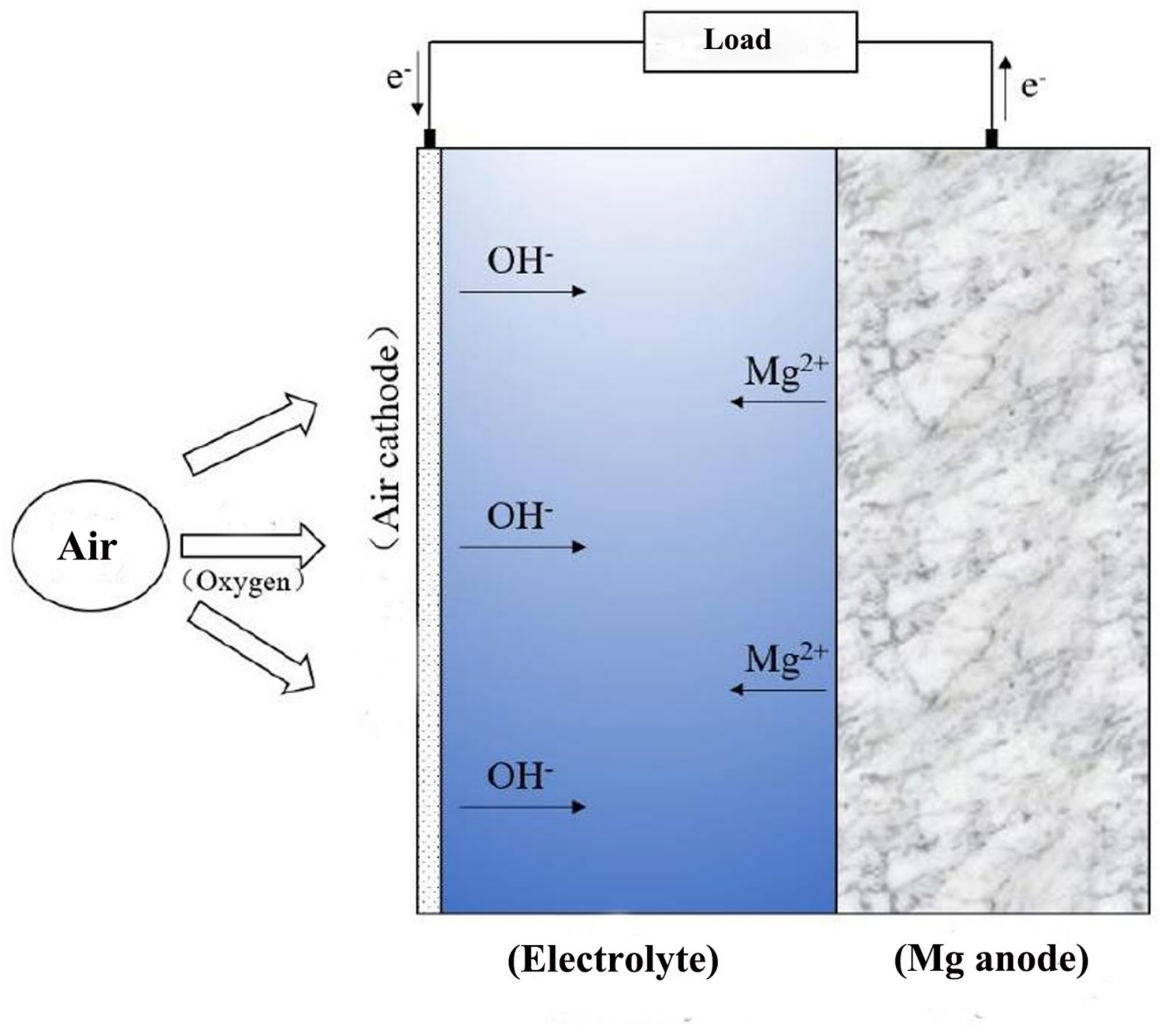
Schematic diagram of a magnesium-air battery^7^.

Against the backdrop of ever-increasing global energy demands, the development of novel energy storage technologies that simultaneously achieve high efficiency, environmental friendliness, and sustainability has emerged as a central research focus^1^. In response to escalating environmental pollution and energy crises, particularly the challenges posed by global warming and ecological degradation, energy storage systems have become a key research area as they play a pivotal role in energy conversion and storage^2^. Currently, while mature energy storage technologies such as lithium-ion batteries, lead-acid batteries, and nickel-metal hydride batteries are widely deployed, they still face challenges including high costs, material scarcity, and inadequate environmental sustainability^3^. Consequently, there is an urgent need to develop energy storage systems featuring higher energy density, abundant resources, low cost, and eco-friendly characteristics. Metal-air batteries, as an emerging battery technology, have attracted significant attention since their inception in the 1970s. These batteries utilize oxygen from the air as the cathode active material, which reacts with a metal anode to generate electricity. This battery system offers a simple design and readily available materials. Magnesium, being one of the most abundant metals in the Earth’s crust, possesses notable advantages including low cost, excellent recyclability, and environmental friendliness. Compared to other metal-air batteries, magnesium-air batteries exhibit superior theoretical energy density (6.8 kW h kg^−1^), Faraday capacity (2200 mA h g^−1^), and theoretical discharge voltage (3.1 V)^4^. These characteristics enable magnesium-air batteries to theoretically achieve both high voltage output and large specific capacity, making them particularly promising for applications in portable electronic devices, unmanned aerial vehicles (UAVs), and electric transportation systems. Furthermore, in comparison with lithium-air and zinc-air batteries, magnesium-air batteries feature cost-effective anode materials with abundant reserves. This advantage circumvents issues related to lithium resource scarcity and price volatility while simultaneously reducing environmental pollution during battery production and recycling processes^5^. As an energy storage system combining high energy density, low cost, and environmental benefits, magnesium-air batteries demonstrate broad application prospects. With continuous technological advancements, they are expected to play a significant role in future energy storage systems^6^. A schematic diagram of the magnesium-air battery structure is shown in Fig. 1.

However, despite these advantages, magnesium-air batteries still face several technical challenges in practical applications^8,9^. The magnesium metal exhibits significant self-corrosion in electrolytes, leading to decreased open-circuit voltage and reduced energy output efficiency. As demonstrated by Deng et al.^10^, during discharge, secondary phases exhibit cathodic characteristics relative to the magnesium matrix. The preferential dissolution of the surrounding magnesium matrix causes these secondary phases to detach from the anode substrate. Furthermore, secondary phases and impurity particles can induce localized and non-uniform anode dissolution, which further damages the magnesium matrix and promotes its detachment. This phenomenon of matrix and secondary phase detachment is termed the “Chunk Effect” (CE), which leads to rapid battery capacity decay, reduced anode efficiency, and significant deterioration of discharge performance. Another critical limitation lies in the relatively low catalytic activity of the oxygen reduction reaction (ORR) at the cathode, which substantially restricts the overall performance of magnesium-air batteries. To address these challenges, recent research efforts have primarily focused on three key aspects: electrode material optimization, electrolyte development, and catalyst design^11–13^.

Alloying magnesium with additional elements represents an effective approach to enhance the corrosion resistance and electrochemical performance of magnesium anodes^14^. This alloying strategy promotes the exfoliation of oxidation products during discharge, thereby reactivating the anode surface^15^. Bismuth (Bi), a Group VA element in the sixth period of the periodic table, possesses a high hydrogen evolution overpotential and has been demonstrated to improve the corrosion resistance of magnesium alloys. During discharge, Bi effectively suppresses the hydrogen evolution reaction (HER), consequently enhancing the discharge efficiency of magnesium anodes^16,17^. Chen et al.^18^ investigated the effect of optimal Bi addition on the discharge performance of magnesium alloy anodes in air batteries. Their results revealed that the Mg-0.5Bi alloy exhibits superior electrochemical behavior and discharge stability, attributed to its lower open-circuit potential (OCP) and the formation of a loosely adherent discharge product layer. Moreover, this alloy significantly mitigates the “Chunk Effect,” highlighting its promising potential for magnesium-air battery anode applications. The alloying of rare earth (RE) elements, including Gd, Y, Nd, Ce, and La, has been extensively studied for their impact on the discharge performance of magnesium-air battery anodes^19–23^. These RE elements facilitate the precipitation of RE-containing phases and refine the alloy microstructure. By reducing secondary phase formation and promoting grain refinement, RE elements enhance the discharge performance of magnesium alloy anodes.

However, despite these advancements, several research gaps remain. Firstly, while the individual effects of Bi and certain RE elements are recognized, the synergistic mechanism between Bi and specific RE elements, particularly Nd, in forming unique intermetallic compounds (e.g., NdBi) and its consequent impact on microstructure control and discharge product exfoliation is not fully understood. Recent studies continue to explore RE-containing anodes. For instance, Liu et al. developed Ca and Nd co-doped AZ61 anodes, demonstrating enhanced discharge performance^24^. Similarly, research on Mg-Sn-based systems highlights the ongoing pursuit of cost-effective anode materials^25^. Secondly, many reported high-performance anode materials rely on high-cost RE additions, creating a demand for developing cost-effective alternatives with lean alloy designs. Thirdly, a systematic comparison of the efficiency of different RE elements (Gd, Y, Nd, Ce, La) when combined with a fixed Bi content in a homogenized state is lacking.

To date, research on the synergistic effects of trace rare earth (RE) elements and bismuth (Bi) on the performance of magnesium-air battery anodes remains limited. To address this knowledge gap, this study fabricated a series of homogeneous Mg-1Bi-0.5X (X = Gd, Y, Nd, Ce, La) alloys. Through systematic investigations of the alloys’ microstructure, electrochemical properties, and discharge performance, we aim to elucidate the synergistic mechanisms between RE elements and Bi. This work is expected to provide new insights and theoretical foundations for developing high-performance magnesium anode materials.

## Experimental materials and methods

### Alloy fabrication

The alloys were prepared using industrial pure Mg (99.9 wt%), pure Bi (99.9 wt%), and master alloys including Mg-25% Gd, Mg-25% Y, Mg-25% Nd, Mg-30% Ce, and Mg-25% La to fabricate Mg-1Bi-0.5X (X = Gd, Y, Nd, Ce, La) alloys. To eliminate compositional segregation in the as-cast alloys, the obtained ingots were homogenized at 400 °C for 12 h in a box-type resistance furnace followed by water quenching. The chemical compositions of the homogenized alloys were verified by inductively coupled plasma optical emission spectrometry (ICP-OES), with the measured compositions listed in Table 1.

**Table 1:** The actual chemical composition of the alloys.

Alloys	Actual chemical composition (wt%)						
	Mg	Bi	Nd	Y	Gd	Ce	La
Mg-1Bi-0.5Nd	Bal.	0.83	0.45	–	–	–	–
Mg-1Bi-0.5Y	Bal.	0.82	–	0.58	–	–	–
Mg-1Bi-0.5Gd	Bal.	0.94	–	–	0.53	–	–
Mg-1Bi-0.5Ce	Bal.	0.84	–	–	–	0.56	–
Mg-1Bi-0.5La	Bal.	0.70	–	–	–	–	0.48

### Experimental methods

The homogenized Mg-1Bi-0.5X (X = Gd, Y, Nd, Ce, La) alloys were used for the following microstructural characterization and performance tests. Microstructural characterization was performed using X-ray diffraction (XRD), optical microscopy (OM), and field-emission scanning electron microscopy (FE-SEM) equipped with energy-dispersive X-ray spectroscopy (EDS). XRD analysis was conducted on a Rigaku SmartLab 9 kW diffractometer with Cu Kα radiation (λ = 0.15406 nm). The scans were performed in the 2θ range of 20° to 90° with a step size of 0.02° and a scanning speed of 5°/min. For OM observation, the samples were mechanically ground with SiC papers up to 5000 grit, polished with diamond paste, and then etched with a solution of 4 vol% nitric acid in ethanol. The metallographic microstructure was examined using a Nikon Eclipse MA200 optical microscope. FE-SEM observations and EDS analysis were carried out on a Thermo Scientific Apreo 2 microscope operating at 15 kV. The EDS point analysis and elemental mapping were performed to identify the composition and distribution of the secondary phases.

Electrochemical measurements were conducted at room temperature using a standard three-electrode system (CHI660E electrochemical workstation) with a 3.5 wt% NaCl solution as the electrolyte. The three-electrode system consisted of a platinum sheet as the counter electrode, a saturated calomel electrode (SCE) as the reference electrode, and the magnesium alloy sample with an exposed area of 1 cm^2^ as the working electrode.

The working electrode was immersed in the solution for 3600 s to attain a stable open-circuit potential (OCP). Electrochemical impedance spectroscopy (EIS) was subsequently performed over a frequency range of 100 kHz to 0.01 Hz with a sinusoidal potential amplitude of 5 mV versus the OCP. The potentiodynamic polarization curves were measured at a scanning rate of 0.5 mV/s. Furthermore, the acquired EIS data were fitted to the equivalent circuit model using the specialized software ZView (Scribner Associates Inc.) to extract the detailed interface parameters. All electrochemical tests were repeated at least three times using independently prepared samples to ensure reproducibility.

The electrochemical discharge performance of the magnesium alloy anodes was evaluated using a custom-built magnesium-air battery testing system with 3.5 wt% NaCl electrolyte as the aqueous electrolyte. Galvanostatic discharge tests were conducted at various current densities ranging from 2.5 to 20 mA cm^−2^ for a duration of 10 h. Following the discharge tests, the discharge products were effectively removed via ultrasonic cleaning in a chromic acid solution (100 g CrO_3_ + 5 g AgNO_3_ + 500 mL H_2_O) to ensure accurate characterization of the electrode surface. The discharge performance was systematically assessed by calculating three key parameters: (i) average discharge voltage, (ii) anodic efficiency, and (iii) specific capacity. Notably, the anodic efficiency and specific capacity were determined according to the calculation method proposed by Ma et al.^26^, which incorporates the theoretical mass loss under applied current conditions to provide more reliable electrochemical evaluation.

After EIS measurements, the samples were carefully extracted from the electrolyte, rinsed with distilled water, and dried in air. The surface morphology and composition of the post-EIS samples were examined using scanning electron microscopy (SEM) coupled with energy-dispersive X-ray spectroscopy (EDS) to characterize the corrosion products and surface changes induced by the electrochemical processes.

## Results

### Microstructural characteristics

**Fig. 2:**
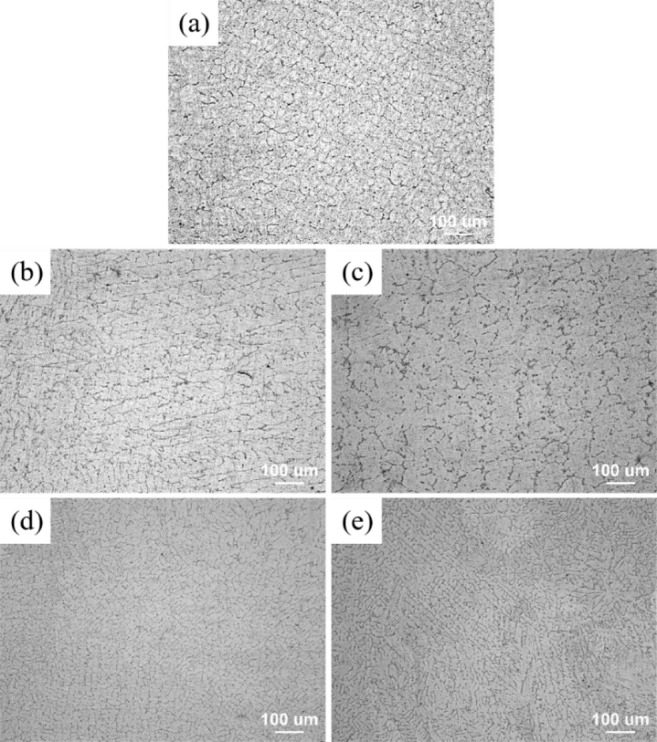
Optical microscopy (OM) images of the homogenized alloys at 100× magnification: (**a**) Mg-1Bi-0.5Gd, (**b**) Mg-1Bi-0.5Y, (**c**) Mg-1Bi-0.5Nd, (**d**) Mg-1Bi-0.5Ce, (**e**) Mg-1Bi-0.5La.

After homogenization treatment, the five Mg-1Bi-0.5X (X = Gd, Y, Nd, Ce, La) alloys exhibited distinct grain structures and varying grain sizes, as shown in the optical microscopy (OM) images at 100× magnification in Fig. 2. We acknowledge that the contrast of some OM images is not ideal due to the low etching response of certain homogenized alloys; however, the grain boundaries were clearly identifiable during the actual measurement process, and the reported grain size data are reliable.

**Fig. 3:**
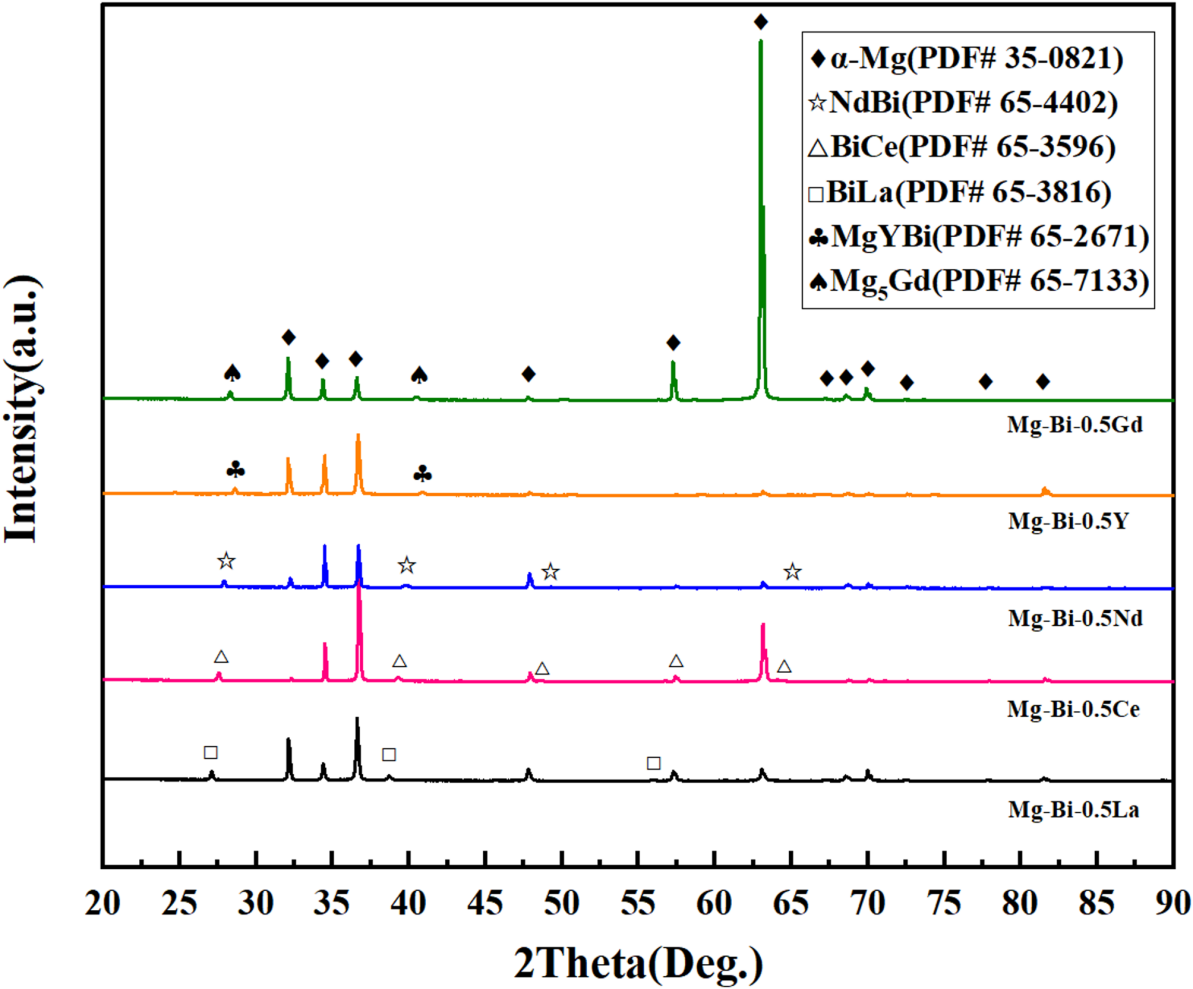
X-ray diffraction (XRD) patterns of the homogenized Mg-1Bi-0.5X (X = Gd, Y, Nd, Ce, La) alloys.

The composition and distribution of phases in the alloy directly influence its electrochemical and discharge performance. To identify the phase constituents, XRD analysis was performed on the alloys, as shown in Fig. 3. The diffraction peaks were identified by referring to the standard powder diffraction files (PDF) from the International Centre for Diffraction Data (ICDD). The primary α-Mg matrix was matched with PDF# 35–0821, while the secondary phase NdBi was consistent with PDF# 65-4402. Notably, none of the five homogenized alloys exhibited diffraction peaks corresponding to the Mg_3_Bi_2_ phase (PDF# 28–0441), which may be attributed to either the low Bi content or the preferential formation of other secondary phases between Bi and the alloying elements.

The average grain size (crystallite size) of the α-Mg matrix in the homogenized alloys was estimated from the X-ray diffraction data using the Scherrer equation^27^:

$$\:D=\frac{K\lambda\:}{\beta\:\mathrm{c}\mathrm{o}\mathrm{s}\theta\:}$$

where $\:D$ is the average crystallite size (nm), $\:K$ is the Scherrer constant (0.89), $\:\lambda\:$ is the X-ray wavelength (0.15406 nm for Cu Kα radiation), $\:\beta\:$ is the full width at half maximum (FWHM in radians) of the diffraction peak after correcting for instrumental broadening, and $\:\theta\:$ is the Bragg angle. The (101) peak of α-Mg at approximately 36.6° was selected for calculation due to its high intensity and clear separation from neighboring peaks. The instrumental broadening was determined using a standard LaB6 sample. The calculated average crystallite sizes for the α-Mg phase in the Mg-1Bi-0.5Nd, Mg-1Bi-0.5Gd, Mg-1Bi-0.5Y, Mg-1Bi-0.5Ce, and Mg-1Bi-0.5La alloys were approximately 557 μm, 1244 μm, 1310 μm, 782 μm, and 1183 μm, respectively. These nanoscale crystallite dimensions, significantly finer than the microscopic grain sizes measured by OM (Fig. 1), contribute to the high density of grain boundaries, which is beneficial for both corrosion resistance and discharge activity.

**Fig. 4:**
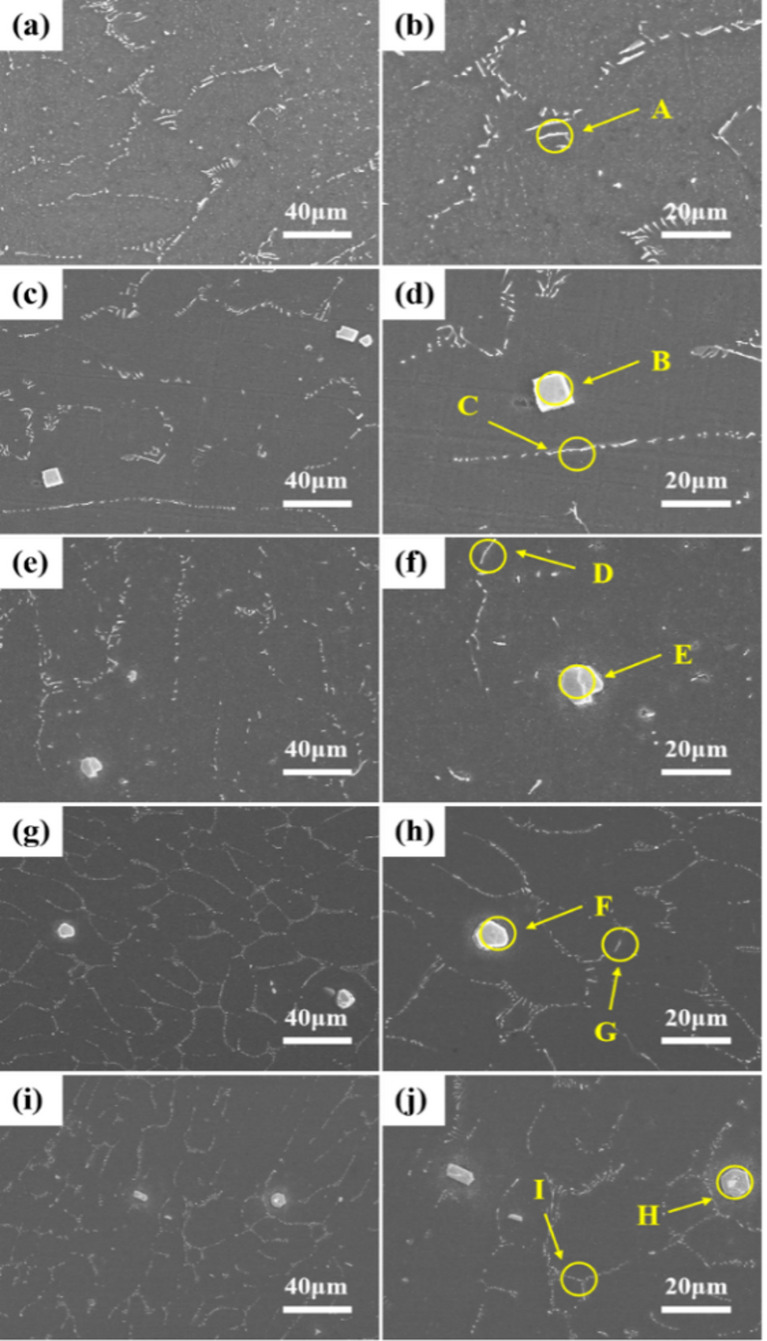
Scanning electron microscopy (SEM) images of the homogenized alloys showing the morphology and distribution of secondary phases: (**a**,**b**) Mg-1Bi-0.5Gd alloy. (**c**,**d**) Mg-1Bi-0.5Y alloy. (**e**,**f**) Mg-1Bi-0.5Nd alloy. (**g**,**h**) Mg-1Bi-0.5Ce alloy. (**i**,**j**) Mg-1Bi-0.5La alloy.

To further investigate the phase composition and distribution in the alloys, scanning electron microscopy (SEM) was employed to analyze the microstructural morphology, with the results presented in Fig. 4, which includes SEM images at 2500x magnification (left) and 5000x magnification (right). Energy-dispersive X-ray spectroscopy (EDS) was subsequently utilized to characterize the constituent phases, as summarized in Table 2. Microstructural observations revealed that the secondary phases in all five alloys exhibited blocky and rod-like morphologies with a semi-continuous network distribution. EDS point analysis (Table 2) confirmed the chemical composition of these phases. Quantitative analysis demonstrated that the area fractions of secondary phases in Mg-1Bi-0.5X (X = Gd, Y, Nd, Ce, La) alloys were 5.2%, 6.3%, 1.3%, 3.3%, and 4.6%, respectively.

**Table 2:** EDS analysis for (a) Mg-1Bi-0.5Gd alloy; (b) Mg-1Bi-0.5Y alloy; (c) Mg-1Bi-0.5Nd alloy; (d) Mg-1Bi-0.5Ce alloy; (e) Mg-1Bi-0.5La alloy.

Marked points	Chemical Composition /at%							Phase composition
	Mg	Bi	Gd	Y	Nd	Ce	La	
(a)								
A	86.32	0	13.68					Mg_5_Gd
(b)								
B	55.24	23.14		21.62				MgYBi
C	87.47	6.16		6.37				MgYBi
(c)								
D	94.86	2.61			2.53			NdBi
E	23.29	39.85			36.86			NdBi
(d)								
F	24.29	38.86				36.85		BiCe
G	94.23	3.04				2.72		BiCe
(e)								
H	25.10	38.30					36.60	BiLa
I	94.08	3.01					2.91	BiLa

### Electrochemical performance

**Fig. 5:**
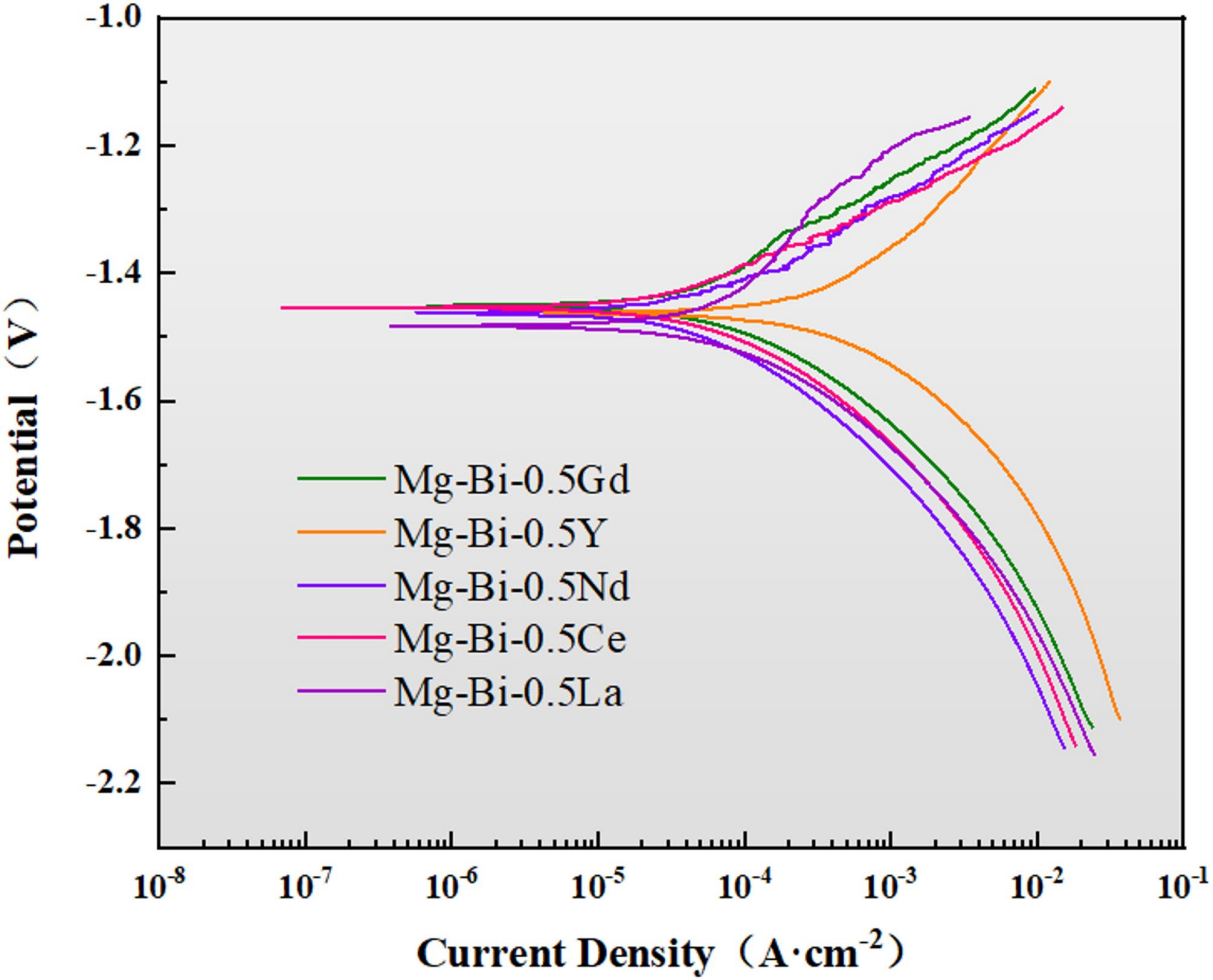
Potentiodynamic polarization curves of the homogenized Mg-1Bi-0.5X (X = Gd, Y, Nd, Ce, La) alloys measured in a 3.5 wt% NaCl solution.

The potentiodynamic polarization curves of the five homogenized alloys in 3.5 wt% NaCl solution are presented in Fig. 5. The cathodic branch of the polarization curves corresponds to the hydrogen evolution reaction, while the anodic branch represents the dissolution of the α-Mg matrix. The fitted corrosion parameters are summarized in Table 3. Among the tested alloys, homogenized Mg-1Bi-0.5Nd exhibited the most positive corrosion potential (-1.46 V) and the lowest corrosion current density (27.03 µA cm^−2^), indicating its superior corrosion resistance.

**Table 3:** Corrosion parameters from polarization curves of the homogeneous alloy in 3.5wt.%NaCl solution.

Alloys	E_corr_ (V)	I_corr_ (µA cm^− 2^)	*P*_i_ (mm year^− 1^)
Mg-1Bi-0.5Gd	-1.44 ± 0.02	64.90 ± 5.2	1.48 ± 0.012
Mg-1Bi-0.5Y	-1.46 ± 0.03	216.60 ± 18.5	4.95 ± 0.42
Mg-1Bi-0.5Nd	-1.46 ± 0.02	27.03 ± 2.15	0.62 ± 0.15
Mg-1Bi-0.5Ce	-1.45 ± 0.02	37.03 ± 3.1	0.85 ± 0.07
Mg-1Bi-0.5La	-1.48 ± 0.03	50.80 ± 4.25	1.16 ± 0.1

The corrosion performance of the developed Mg-1Bi-0.5Nd alloy was compared with typical anode materials reported in the literature to highlight its advancement. For instance, the commercial AZ31 alloy, a commonly used benchmark, exhibits a corrosion current density (I_corr) in the range of 80–150 µA cm^−2^ in 3.5% NaCl solution^28,29^. The Icorr of Mg-1Bi-0.5Nd alloy (27.03 µA cm^−2^) is significantly lower, representing a ~ 70% reduction, which underscores its enhanced corrosion resistance. This improvement can be primarily attributed to the refined grain structure and the unique semi-continuous network of the NdBi phase, which homogenizes the corrosion process and mitigates severe localized attack.

Furthermore, compared to other recently developed Mg-Bi based anodes, the alloy demonstrates superior properties. Nivedhitha et al.^30^ reported that an extruded Mg-0.5Bi alloy achieved an I_corr of approximately 45 µA cm^−2^. The addition of 0.5% Nd in the Mg-1Bi-0.5Nd alloy further reduced the I_corr to 27.03 µA cm^−2^. This synergistic effect between Bi and Nd is crucial: Bi elevates the hydrogen evolution overpotential, while Nd promotes the formation of a more protective surface film and refines the microstructure, as confirmed by our EIS results and microstructural analysis. The overall corrosion resistance ranking was established as: Mg-1Bi-0.5Nd > Mg-1Bi-0.5Ce > Mg-1Bi-0.5La > Mg-1Bi-0.5Gd > Mg-1Bi-0.5Y. The exceptionally poor performance of Mg-1Bi-0.5Y is likely due to the formation of coarse cathodic MgYBi phases (Table 2, Points B and C), which exacerbated micro-galvanic corrosion. Notably, *E*_corr_ reflects thermodynamic activity, with more negative values generally indicating enhanced activation capability during the initial discharge stage^31^.

**Fig. 6:**
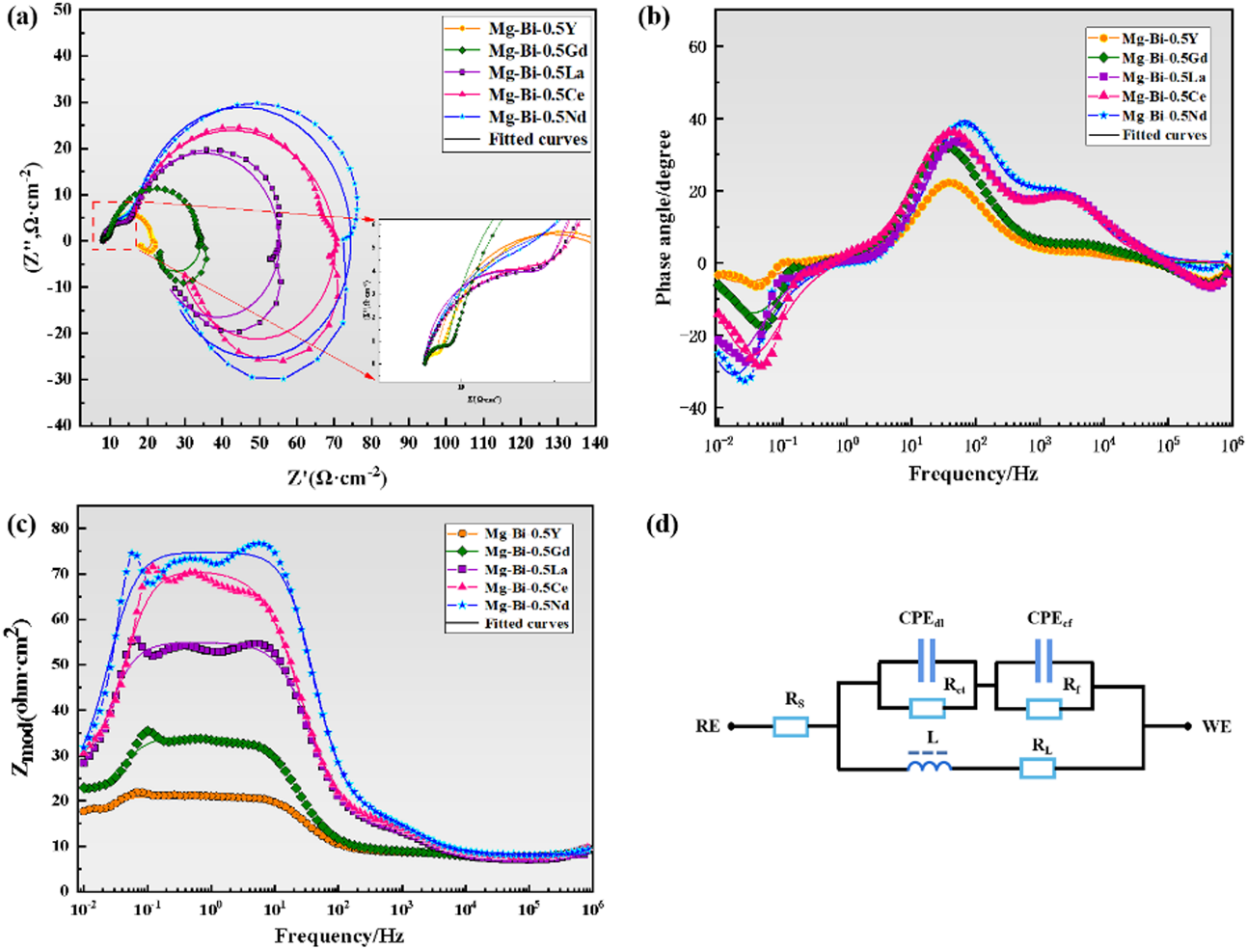
EIS spectra of five alloys. (**a**) Nyquist plot; (**b**) bode phase angle plot; (**c**) impedance magnitude plot; (**d**) equivalent circuit diagram.

The electrochemical impedance spectra (EIS) of the five homogenized alloys were measured at the open-circuit potential with an amplitude of 5 mV over the frequency range from 100 kHz to 0.01 Hz. The obtained data were fitted using the equivalent electrical circuit (EEC) shown in Fig. 6d, and the detailed fitted parameters are listed in Table 4. While R_s_ represents the solution resistance and was consistently low for all alloys due to the conductive NaCl electrolyte, and R_ct_ is a direct indicator of charge transfer resistance at the metal/electrolyte interface, a deeper analysis of the other parameters provides further crucial insights into the corrosion mechanisms.

The constant phase elements (CPE_dl_ and CPE_f_) were used instead of ideal capacitors to account for the non-ideal behavior of the electrical double layer and surface film, respectively, often caused by surface inhomogeneity, roughness, or porosity. The ‘n’ values for CPE_dl_ range from 0.89 to 0.99. The Mg-1Bi-0.5Nd alloy exhibits an n_1_ value closest to 1 (0.99), indicating that its double-layer behavior is nearly capacitive. This suggests a relatively homogeneous and stable surface, which is consistent with its fine-grained microstructure and uniform distribution of the NdBi phase. In contrast, the lower n_1_ value for Mg-1Bi-0.5Ce (0.89) suggests greater surface inhomogeneity, likely due to a less protective film.

The film resistance (R_f_) and film capacitance (CPE_f_) parameters are associated with the layer of corrosion products formed on the surface. The Mg-1Bi-0.5Nd and Mg-1Bi-0.5Ce alloys, which have the highest R_ct_ values, also show moderate R_f_ values. This indicates that the protective effect stems not from a thick, resistive barrier film (which would have a very high R_f_), but rather from a dense, effective film that profoundly inhibits the charge transfer process at the underlying metal interface. The significantly lower R_f_ value for Mg-1Bi-0.5Y (12.52 Ω cm^2^) aligns with its poor corrosion resistance, indicating a highly defective and non-protective surface film.

Perhaps the most intriguing parameter is the inductance (L and R_L_), which appears as a low-frequency inductive loop. This feature is frequently associated with the relaxation of adsorbed species on the metal surface, such as Mg^+^ intermediates, or the initiation of localized pitting corrosion. A lower R_L_ value, as observed for Mg-1Bi-0.5Nd (20.73 Ω cm^2^), suggests a faster desorption rate of these adsorbed species or corrosion products^32^. This facilitates the re-exposure of fresh active surface area to the electrolyte, which is critically important for sustaining high discharge activity in magnesium-air batteries by preventing passivation. The large inductance value (L = 452.2 H cm^2^) for Mg-1Bi-0.5Nd further confirms strong adsorption processes and excellent product detachment capabilities, which is consistent with its superior discharge stability and high anode efficiency as demonstrated by the impedance and discharge results. Conversely, the high R_L_ and lower L value for Mg-1Bi-0.5Y imply sluggish surface processes, leading to accumulation of corrosion products and rapid performance degradation.

In summary, the comprehensive EIS analysis reveals that the superior performance of the Mg-1Bi-0.5Nd alloy is not just due to a high R_ct_, but is a result of a synergistic combination of properties: a homogeneous surface (*n* ~ 1), an effective charge-transfer inhibiting film, and exceptionally facile adsorption/desorption kinetics (low R_L_, high L) that ensure ongoing electrochemical activity.

The electrochemical reactions of magnesium alloys in aqueous electrolytes typically involve the anodic dissolution of magnesium and the cathodic evolution of hydrogen, leading to the formation of magnesium hydroxide:

1 $${\mathrm{Mg}} \to {\mathrm{Mg}}^{2+} + 2{\mathrm{e}}^{-}$$

2 $$2{\mathrm{H}}_{2}{\mathrm{O}}+2{\mathrm{e}}^{-} \to {\mathrm{H}}_{2} + 2{\mathrm{OH}}^{-}$$

3 $${\mathrm{Mg}}^{2+} + 2{\mathrm{OH}}^{-} \to {\mathrm{Mg(OH)}}_{2}$$

Or, as the overall reaction:

4 $${\mathrm{Mg}}+2{\mathrm{H}}_{2}{\mathrm{O}} \to {\mathrm{Mg(OH)}}_{2}+{\mathrm{H}}_{2}$$

Based on the Nyquist plot and the overall corrosion reaction of the alloy in a 3.5 wt% NaCl solution, the equivalent electrical circuit (EEC) is illustrated in Fig. 6d. In this circuit, the primary parameters include the charge transfer resistance (R_ct_), the electrolyte resistance (R_s_), and the double-layer capacitance (CPE_dl_) at the substrate/film interface. The film resistance (R_f_) and film capacitance (CPE_f_) are also represented. The inductive loop is characterized by L and R_L_, where a lower RL value (20.73 Ω cm^2^) indicates a rapid desorption rate of the corrosion product film, thereby increasing the active surface area of the magnesium anode in air batteries^33^. The impedance data were fitted, and the results are summarized in Table 4. Due to the high conductivity of the NaCl solution, the R_s_ values are nearly identical and relatively small. The corrosion resistance of the alloys was evaluated based on the Rct values—generally, a higher R_ct_ corresponds to better corrosion resistance. The Mg-1Bi-0.5Nd alloy exhibits a significantly higher R_ct_ value compared to the other two alloys, indicating superior corrosion resistance. This can be attributed to the protective effect of the corrosion product film on the magnesium substrate. The addition of 0.5% Nd, compared to the other four alloying elements, promotes the formation of a denser oxide film during corrosion, which impedes electron transfer and slows down the corrosion process. Consequently, based on the R_ct_ values, the corrosion resistance of the five homogenized alloys can be ranked in descending order as follows: Mg-1Bi-0.5Nd > Mg-1Bi-0.5Ce > Mg-1Bi-0.5La > Mg-1Bi-0.5Gd > Mg-1Bi-0.5Y.

**Table 4:** Fitting results of EIS measurement.

Alloys	Anodic	CPE_dl_		*R*_ct_	CPE_f_		*R*_f_	*R*_L_	L
	Ω cm^2^	Y_1_/µΩ^−1^ cm^− 2^	*n*_1_	Ω cm^2^	Y_2_/µΩ^−1^ cm^− 2^	*n*_2_	Ω cm^2^	Ω cm^2^	H cm^2^
Mg-1Bi-0.5Gd	7.859 ± 0.15	42.30	0.96	1.155 ± 0.15	489.6	0.92	25.23 ± 2.5	26.07	192.4 ± 25
Mg-1Bi-0.5Y	8.176 ± 0.16	98.96	0.95	0.59 ± 0.08	691.8	0.93	12.52 ± 1.8	34.01	265.3 ± 30
Mg-1Bi-0.5Nd	8.267 ± 0.17	89.6	0.99	57.35 ± 5	51.48	0.85	9.16 ± 1.2	20.73	452.2 ± 40
Mg-1Bi-0.5Ce	8.074 ± 0.16	248.6	0.89	56.27 ± 4.8	17.17	0.96	6.65 ± 1	29.69	280.7 ± 30
Mg-1Bi-0.5La	7.526 ± 0.15	15.81	0.96	47.44 ± 4.5	116.5	0.95	9.927 ± 1.5	20.58	390.6 ± 35

#### Post-mortem characterization of EIS samples

**Fig. 7:**
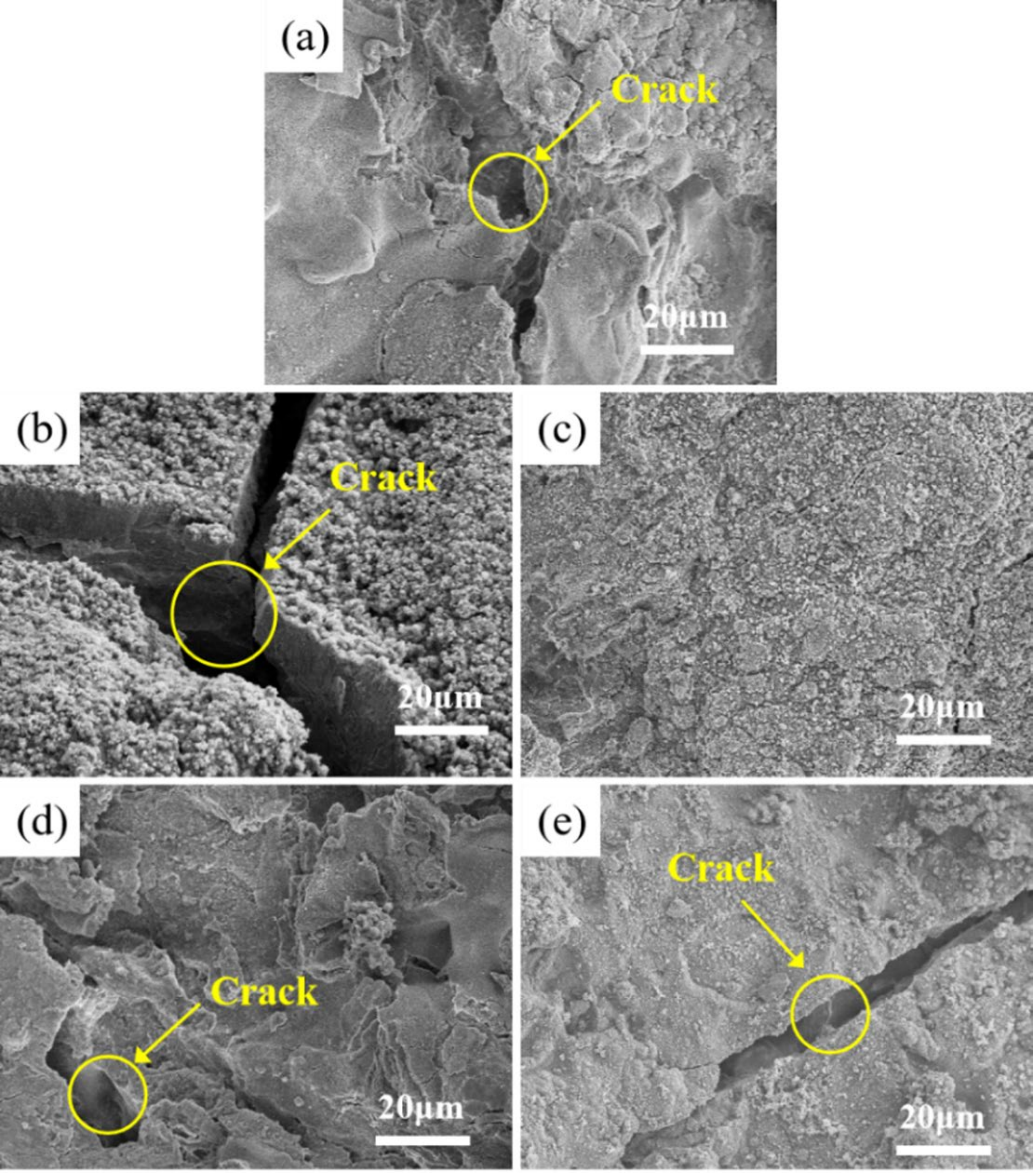
Scanning electron microscopy (SEM) images showing the surface morphology of the homogenized alloys after electrochemical impedance spectroscopy (EIS) testing, with corrosion products retained: (**a**) Mg-1Bi-0.5Gd, (**b**) Mg-1Bi-0.5Y, (**c**) Mg-1Bi-0.5Nd, (**d**) Mg-1Bi-0.5Ce, (**e**) Mg-1Bi-0.5La.

**Fig. 8:**
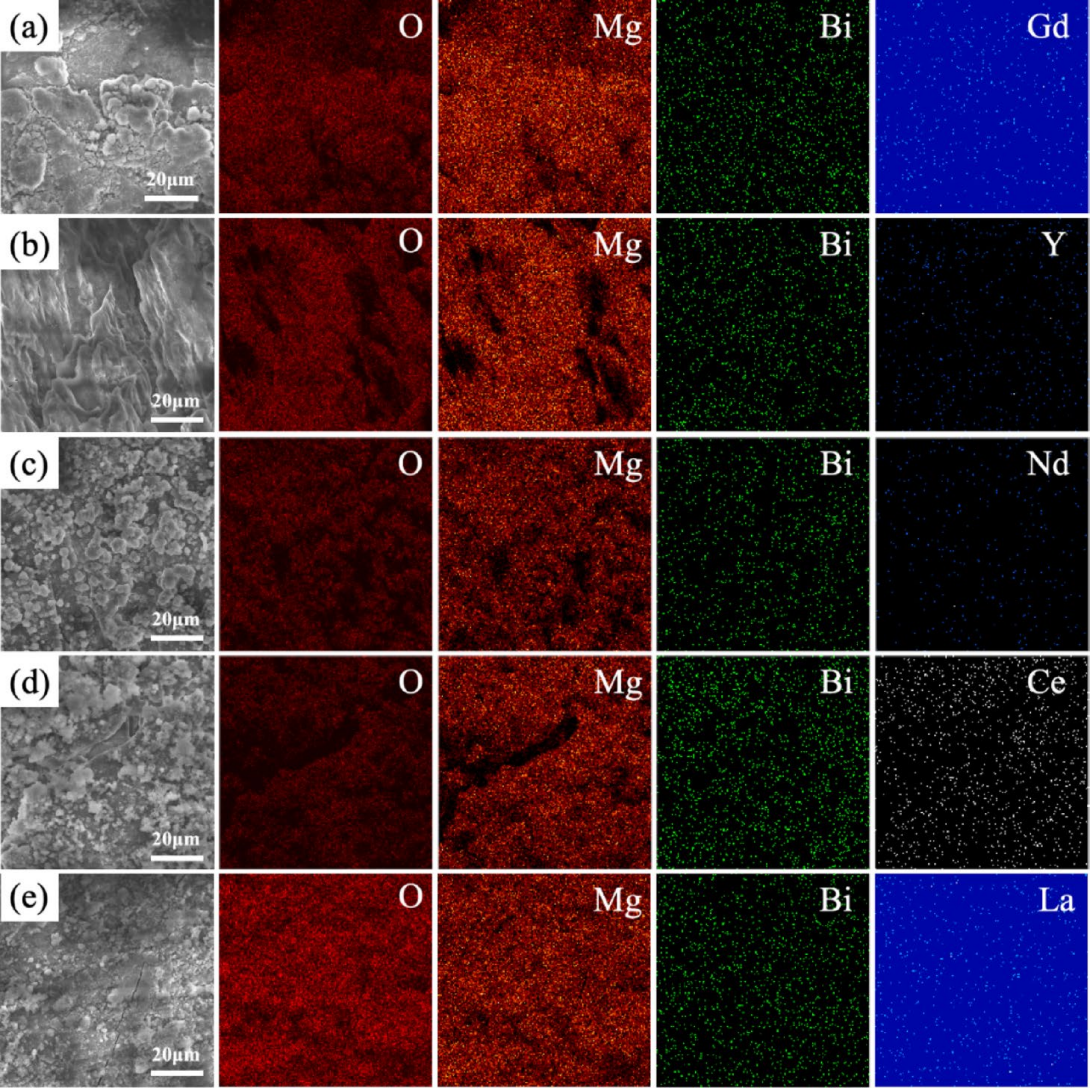
SEM images and corresponding energy-dispersive X-ray spectroscopy (EDS) elemental maps of the corrosion product layers on the homogenized alloys after EIS testing: (**a**) Mg-1Bi-0.5Gd, (**b**) Mg-1Bi-0.5Y, (**c**) Mg-1Bi-0.5Nd, (**d**) Mg-1Bi-0.5Ce, (**e**) Mg-1Bi-0.5La.

The surface morphology of the alloys after EIS testing was investigated using SEM, as shown in Fig. 7. The corrosion product film of the Mg-1Bi-0.5Nd alloy exhibits a spherical morphology distributed across the substrate surface, almost completely covering the alloy. This relatively dense product film, to some extent, hinders the reaction between the alloy and the solution during the corrosion process, thereby slowing down the corrosion rate. In contrast, large and deep corrosion cracks are observed on the surface of the Mg-1Bi-0.5Y alloy, indicating that the corrosion products provide extremely limited protection to the substrate. These extensive and deep cracks suggest that severe corrosion has occurred in the corrosive medium. Furthermore, such cracks increase the effective contact area between the substrate and the corrosive medium, accelerating the corrosion process. Elemental mapping of the corrosion product film, as shown in Fig. 8, detected small amounts of Nd and Bi in the Mg-1Bi-0.5Nd sample, indicating that the alloying elements have been incorporated into the corrosion layer. This dense yet micro-cracked morphology in Mg-1Bi-0.5Nd facilitates electrolyte penetration and product exfoliation, which is consistent with its superior EIS parameters and discharge stability.

While this study demonstrates the superior performance of Mg-1Bi-0.5Nd, several limitations should be acknowledged. First, the scope of alloy compositions was restricted to a fixed Bi content (1 wt%) and limited RE additions (0.5 wt%), leaving other potential ratios unexplored. Second, all electrochemical and discharge tests were conducted in a static 3.5 wt% NaCl solution, which may not fully replicate dynamic or harsh real-world environments^34^.

### Discharge behavior

**Fig. 9:**
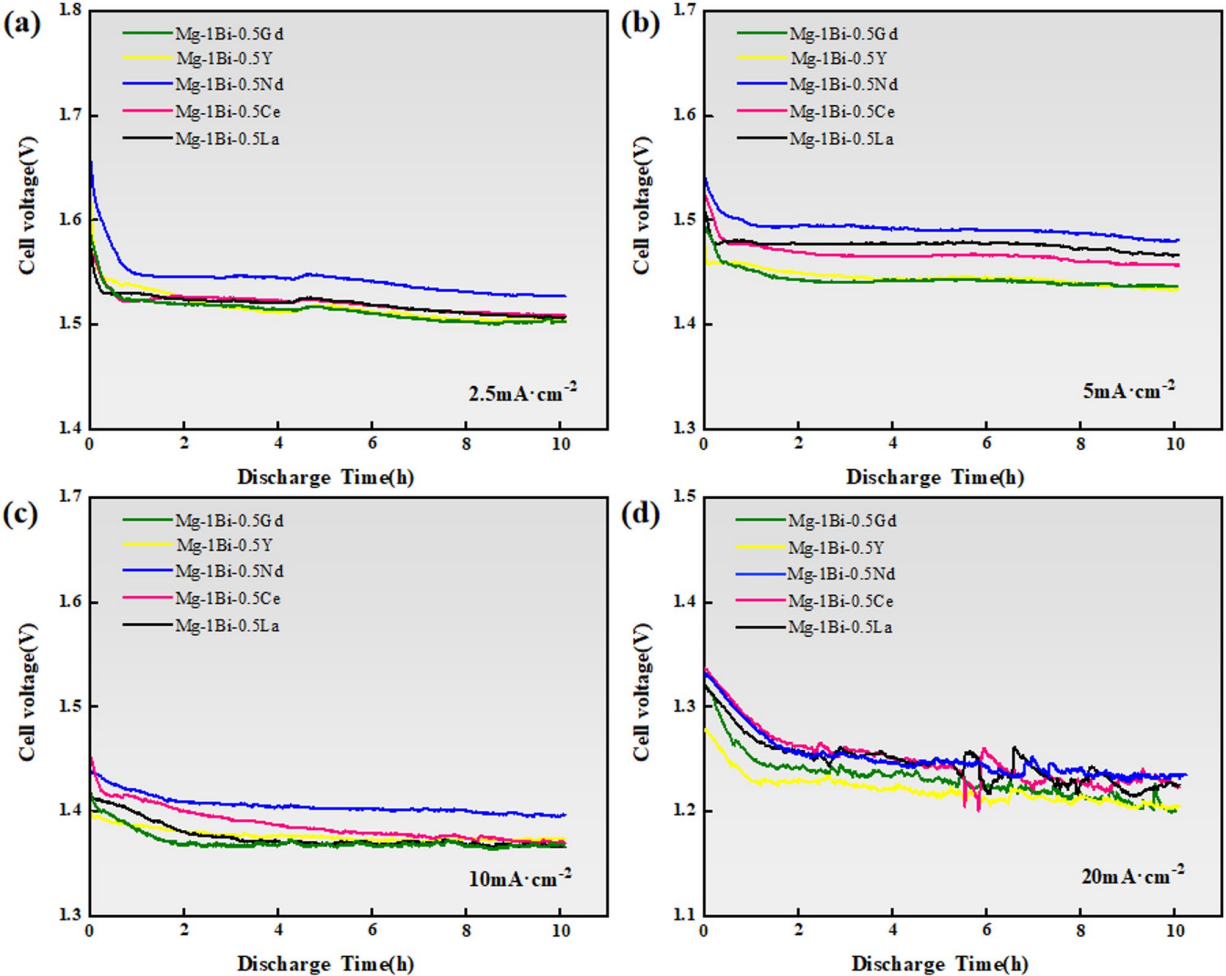
Discharge voltage-time profiles of the homogenized Mg-1Bi-0.5X (X = Gd, Y, Nd, Ce, La) alloy anodes in a magnesium-air battery with 3.5 wt% NaCl electrolyte, measured at different constant current densities for 10 h: (**a**) 2.5 mA cm^−2^, (**b**) 5 mA cm^−2^, (**c**) 10 mA cm^−2^, (**d**) 20 mA cm^−2^.

The discharge performance of homogenized Mg-1Bi-0.5x (x = Gd, Y, Nd, Ce, La) alloy anode materials in a 3.5 wt% NaCl solution was systematically investigated using a self-designed magnesium-air battery testing system. The tests were conducted at current densities ranging from 2.5 to 20 mA cm^−2^ for a duration of 10 h. By analyzing key parameters such as average discharge voltage, anodic efficiency, and specific capacity (Fig. 9), it was observed that all alloys exhibited a significant initial voltage drop at the beginning of discharge, attributed to the rapid accumulation of discharge products on the anode surface. Within the 2.5–10 mA cm^−2^ current density range, the alloys demonstrated stable discharge plateaus, indicating a dynamic equilibrium between the formation and detachment of discharge products. However, at the higher current density of 20 mA cm^−2^, the discharge curves displayed noticeable fluctuations accompanied by voltage decay, primarily due to the slower removal rate of discharge products compared to their generation rate. It is noteworthy that the Mg-1Bi-0.5Nd alloy exhibited the most superior discharge stability under all tested conditions, with the smallest voltage fluctuations.

The superior performance of the Mg-1Bi-0.5Nd alloy can be attributed to several key factors. First, its fine-grained microstructure provides a higher density of grain boundaries, which facilitates ion transport and enhances electrochemical reaction kinetics^35^. Second, the homogeneous distribution of a small amount of secondary phases (1.3%) effectively suppresses micro-galvanic corrosion, preventing localized accumulation of discharge products. Furthermore, electrochemical impedance spectroscopy (EIS) analysis revealed that this alloy exhibits the largest low-frequency inductance value (452.2 H), indicating the strongest desorption capability of discharge products. This significantly increases the active electrode/electrolyte contact area by promoting bubble-mediated product detachment. These synergistic effects collectively enable the Mg-1Bi-0.5Nd alloy to maintain the highest discharge voltage and most stable discharge behavior across a wide range of current densities.

**Fig. 10:**
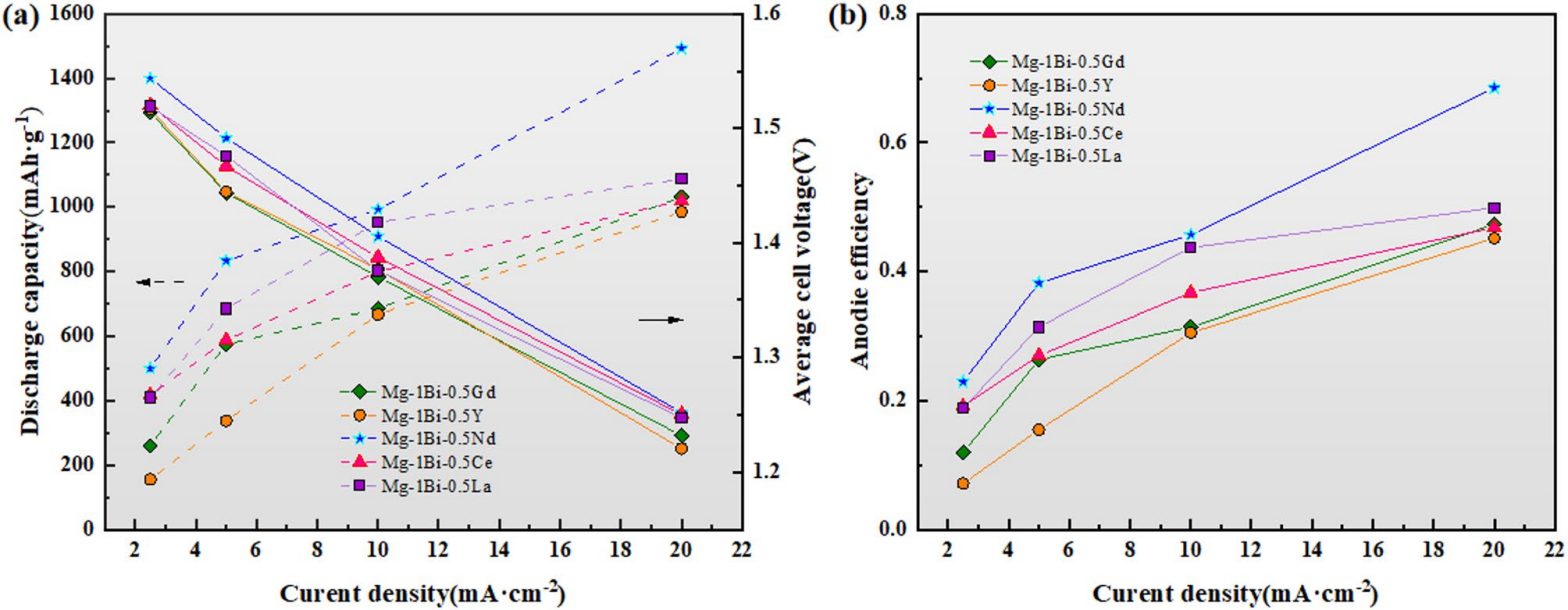
Discharge performance of homogenized Mg-1Bi-0.5x (x = Gd, Y, Nd, Ce, La) alloys as anodes for Mg-air batteries. (**a**) Average discharge voltage and specific capacity, (**b**) Anode efficiency.

Figure 10 presents the discharge performance of homogenized Mg-1Bi-0.5x (x = Gd, Y, Nd, Ce, La) alloys as anode materials for magnesium-air batteries at different current densities, including key parameters such as average discharge voltage, specific capacity, and anodic efficiency (detailed data are provided in Tables 5 and 6). The experimental results demonstrate that as the current density increases, the specific capacity of the alloys shows an upward trend, while both the average discharge voltage and anodic efficiency gradually decrease. Notably, the Mg-1Bi-0.5Nd alloy exhibits the most superior discharge performance under all tested conditions. Taking the Mg-1Bi-0.5Y alloy as an example, at a current density of 2.5 mA cm^−2^, its specific capacity and anodic efficiency are 155.28 mAh g^−1^ and 7.12%, respectively. When the current density increases to 20 mA cm^−2^, these parameters rise to 985.22 mAh g^−1^ and 45.18%, yet remain significantly lower than the corresponding values of the Mg-1Bi-0.5Nd alloy.

The electrochemical behavior of magnesium alloys serving as anodes in air batteries is predominantly governed by two competing reactions: the discharge reaction and the hydrogen evolution reaction. The former facilitates energy output through electron transfer to the external circuit, while the latter generates hydrogen gas via electron capture by hydrogen ions. Experimental observations reveal that as the discharge current density increases, the dissolution rate of the magnesium alloy accelerates significantly, whereas the rate of the hydrogen evolution reaction exhibits relatively minor changes. This disparity results in a greater proportion of electrons being directed toward the external circuit, thereby enhancing the anode’s operational efficiency.

Quantitative analysis reveals an inverse correlation between the actual weight loss of magnesium alloys during discharge and their anodic efficiency. Specifically, the actual weight loss comprises three components: (1) ineffective weight loss caused by hydrogen evolution reaction, (2) effective weight loss participating in electrochemical reactions, and (3) ineffective weight loss resulting from detachment of unreacted substrate (termed the “chunk effect”). Notably, the hydrogen evolution reaction not only directly consumes active material but also alters the electrode surface state, thereby exacerbating the occurrence of the chunk effect. The synergistic action of these two mechanisms constitutes the primary cause for the reduction in anodic efficiency.

Based on the electrochemical test results of the five homogenized alloys, the Mg-1Bi-0.5Y alloy exhibited the poorest corrosion resistance, which generated substantial ineffective weight loss and consequently led to its lower anodic efficiency. Discharge performance analysis of these alloy anodes at different current densities revealed that at 2.5 mA cm^-2^, the Mg-1Bi-0.5Nd alloy demonstrated the highest specific capacity and anodic efficiency among the five alloys. These values showed continuous improvement with increasing current density, reaching peak performance at 20 mA cm^-2^ with outstanding specific capacity (1492.54 mAh g^-1^) and anodic efficiency (68.5%) - the best results among all tested alloys. The discharge performance of the Mg-1Bi-0.5Nd alloy significantly surpasses that of commonly used commercial benchmarks. For instance, the widely studied AZ31 alloy typically reports an anodic efficiency of around 30–40% and a specific capacity of about 700–900 mAh g^−1^ at similar current densities in 3.5% NaCl solution. Our Mg-1Bi-0.5Nd anode demonstrates a ~ 114% improvement in specific capacity compared to AZ31, as mentioned in the abstract. This performance is also superior to many recently developed alloy anodes. Chen et al.^12^ reported that an extruded Mg-0.5Bi alloy achieved an anodic efficiency of approximately 55% at 10 mA cm^−2^. The addition of only 0.5% Nd in the present Mg-1Bi-0.5Nd alloy pushes this efficiency to 68.5% at a much higher current density of 20 mA cm^−2^, highlighting the profound synergistic effect of the Bi-Nd combination. This efficiency is also comparable to or even higher than those of some more expensive RE-rich alloys, such as Mg-Gd-based systems, demonstrating the cost-effectiveness of this lean alloy design. The comprehensive evaluation clearly demonstrates that the Mg-1Bi-0.5Nd alloy possesses optimal discharge performance, making it an ideal anode material for magnesium-air batteries. The performance ranking of the five alloys follows this order: Mg-1Bi-0.5Nd > Mg-1Bi-0.5La > Mg-1Bi-0.5Ce > Mg-1Bi-0.5Gd > Mg-1Bi-0.5Y.

**Table 5:** Specific capacity density and average discharge voltage of five homogenized alloys at different current Densities.

Alloys		Mg-1Bi-0.5Gd	Mg-1Bi-0.5Y	Mg-1Bi-0.5Nd	Mg-1Bi-0.5Ce	Mg-1Bi-0.5La
Discharge capacity (mAh g^− 1^)	2.5 mA cm^− 2^	260.42	155.28	500	416.67	409.84
	5 mA cm^− 2^	574.71	337.84	833.33	588.24	684.93
	10 mA cm^− 2^	684.93	666.67	992.38	800.00	952.38
	20 mA cm^− 2^	1030.93	985.22	1492.54	1020.41	1086.96
Average cell voltage (V)	2.5 mA cm^− 2^	1.51	1.52	1.54	1.52	1.52
	5 mA cm^− 2^	1.44	1.45	1.49	1.47	1.48
	10 mA cm^− 2^	1.37	1.38	1.41	1.39	1.38
	20 mA cm^− 2^	1.23	1.22	1.25	1.25	1.25

**Table 6:** Anode efficiency of five homogenized alloys at different current densities.

Alloys		Mg-1Bi-0.5Gd	Mg-1Bi-0.5Y	Mg-1Bi-0.5Nd	Mg-1Bi-0.5Ce	Mg-1Bi-0.5La
Anodic efficiency	2.5 mA cm^− 2^	0.1195	0.0712	0.2295	0.1912	0.1881
	5 mA cm^− 2^	0.2638	0.1549	0.3824	0.2700	0.3143
	10 mA cm^− 2^	0.3144	0.3057	0.4571	0.3671	0.4371
	20 mA cm^− 2^	0.4732	0.4518	0.6850	0.4683	0.4988

**Fig. 11:**
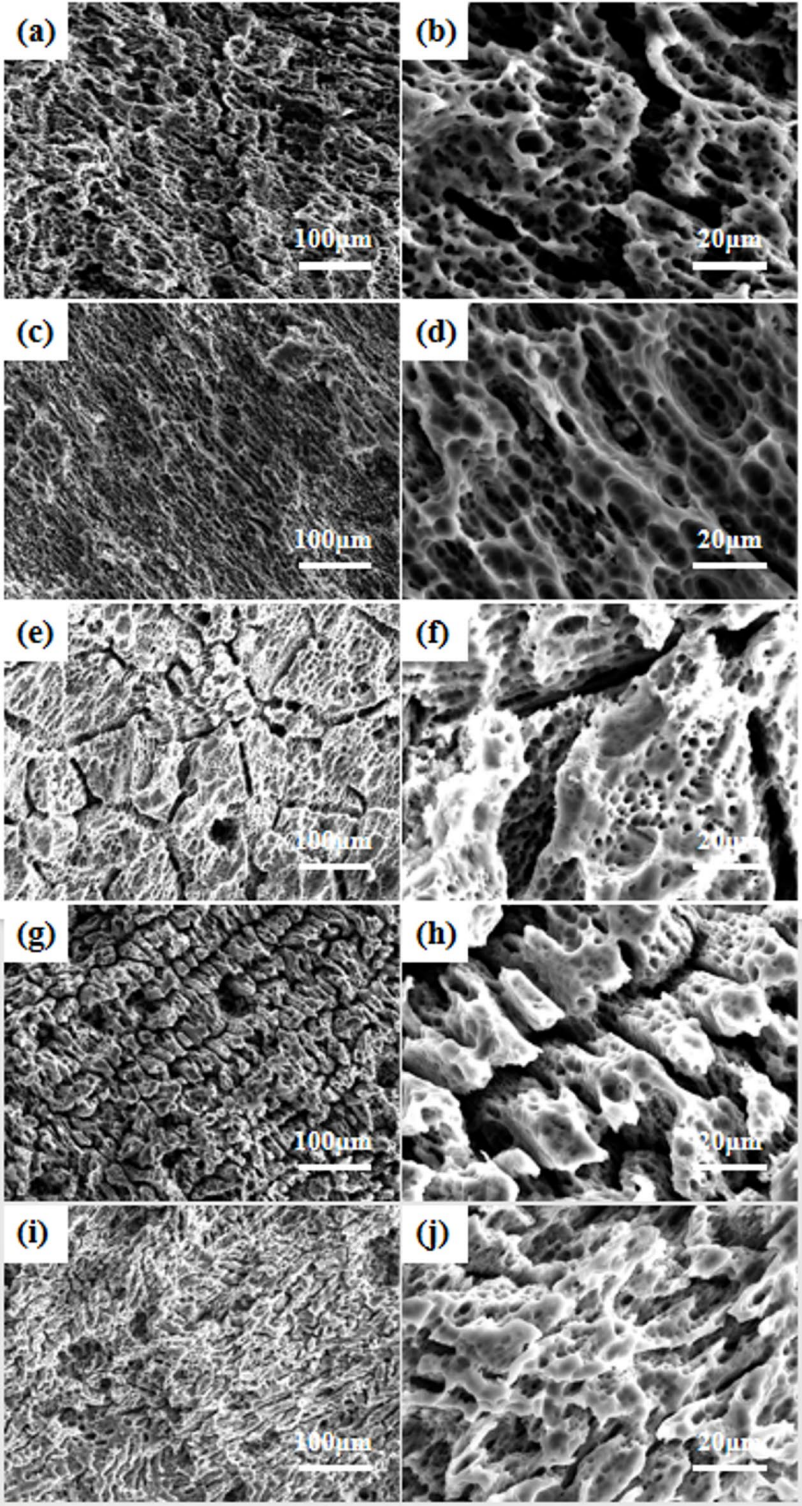
Scanning electron microscopy (SEM) images showing the surface morphology of the homogenized alloy anodes after 10 h of discharge at 2.5 mA cm^−2^ and subsequent removal of the discharge products: (**a**,**b**) Mg-1Bi-0.5Gd, (**c**,**d**) Mg-1Bi-0.5Y, (**e**,**f**) Mg-1Bi-0.5Nd, (**g**,**h**) Mg-1Bi-0.5Ce, (**i**,**j**) Mg-1Bi-0.5La. Images in the left and right columns are at different magnifications to show overall and localized corrosion features, respectively.

Figure 11 presents the SEM morphologies at 1000x magnification of the five homogenized alloys after 10-hour discharge at 2.5 mA cm^−2^ with surface products removed. Comparative analysis reveals significant differences in corrosion morphology among the alloys. Specifically, the Mg-1Bi-0.5Gd, Mg-1Bi-0.5Y, and Mg-1Bi-0.5La alloys exhibit characteristic fishbone-shaped corrosion grooves (Fig. 4a,c,g,i), which are closely associated with their dendritic microstructures and tend to hinder the effective detachment of discharge products. Furthermore, distinct deep pit structures are observed on these alloy surfaces, primarily attributed to localized corrosion induced by hydrogen evolution during the discharge process.

Notably, the Mg-1Bi-0.5Nd alloy demonstrates unique surface morphological characteristics: (1) a relatively smooth corrosion surface that facilitates uniform detachment of discharge products, and (2) a well-distributed micro-crack network that effectively increases the electrode-electrolyte contact area. This distinctive morphology offers dual advantages. On one hand, the smooth surface minimizes the accumulation of discharge products. On the other hand, the micro-crack network maintains sufficient active reaction area during later discharge stages, thereby mitigating voltage drop caused by product buildup. This finding provides a clear explanation for the superior specific capacity and anodic efficiency exhibited by the Mg-1Bi-0.5Nd alloy.

**Fig. 12:**
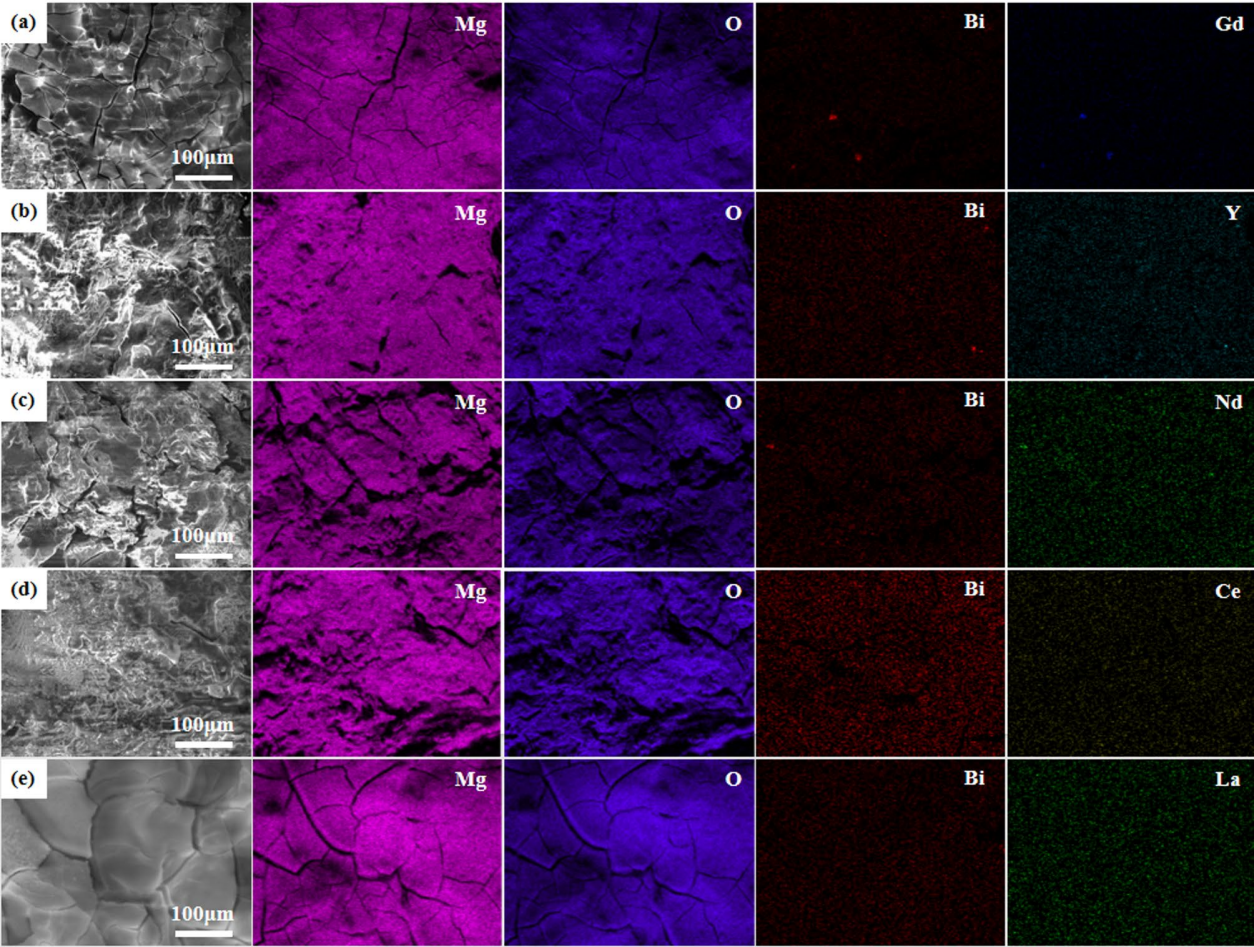
The surface sweep of the five homogeneous alloys at a current density of 2.5 mA cm^-2^ for 10 h. (**a**) Mg-1Bi-0.5Gd, (**b**) Mg-1Bi-0.5Y, (**c**) Mg-1Bi-0.5Nd, (**d**) Mg-1Bi-0.5Ce, (**e**) Mg-1Bi-0.5La.

Figure 12 presents the SEM morphology and EDS mapping results of surface products formed on five homogenized alloys after 10-hour galvanostatic discharge at 2.5 mA cm^− 2^. EDS analysis reveals that all surface products are rich in Mg and O elements (atomic ratio ≈ 1:2), confirming their primary composition as Mg(OH)_2_, which is consistent with previously reported anode products in magnesium-air batteries^36^. Notably, despite similar chemical composition, the products exhibit distinct microstructural characteristics. In contrast to the dense product layers observed on other alloys, the Mg-1Bi-0.5Nd alloy displays a unique loose and porous structure featuring: (1) well-developed crack networks (average width: 2–5 μm) and (2) pronounced lamellar exfoliation features. This distinctive morphology significantly enhances discharge performance through three mechanisms: First, it increases the effective reaction area - the cracks and pores substantially expand the triple-phase boundary between electrode and electrolyte, facilitating charge transfer. Second, it promotes product detachment - hydrogen bubbles generated by the hydrogen evolution reaction (Mg + 2H_2_O → Mg(OH)_2_ + H_2_↑) readily accumulate in the porous structure, causing localized exfoliation when bubble pressure exceeds the bonding strength of the product layer. Third, it maintains continuous reaction - the dynamically renewing surface structure prevents active site passivation that typically occurs with dense product layers.

**Fig. 13:**
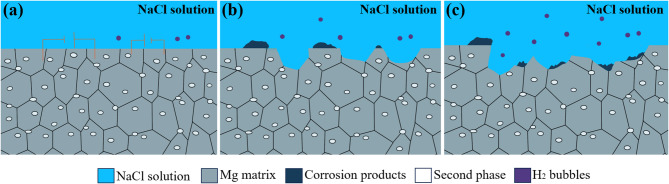
Schematic diagram illustrating the proposed discharge process and microstructural evolution of the Mg-1Bi-0.5Nd alloy anode in a magnesium-air battery: (**a**) Initial stage, (**b**) middle stage, (**c**) late stage.

Figure 13a–c schematically illustrates the microstructural evolution of the Mg-1Bi-0.5Nd alloy during the initial, intermediate, and final stages of discharge, respectively.

During the initial discharge stage (Fig. 10a), the alloy surface is directly exposed to the 3.5 wt% NaCl electrolyte… At this stage, the anodic magnesium dissolution reaction (Reaction 1) occurs rapidly, accompanied by the cathodic oxygen reduction reaction (ORR):

5 $${\mathrm{O}}_{2}+2{\mathrm{H}}_{2}{\mathrm{O}}+{\mathrm{e}}^{-} \to 4{\mathrm{OH}}^{-}$$

The abundant active sites on the electrode surface facilitate fast reaction kinetics, resulting in the highest initial voltage observed during this phase.

The abundant active sites on the electrode surface facilitate fast reaction kinetics, resulting in the highest initial voltage observed during this phase.

In the middle discharge stage (Fig. 10b), discontinuous deposition of Mg(OH)_2_ discharge products (shown as black irregular regions) forms a porous structure. This porous layer enables electrolyte penetration and maintains ion transport channels, thereby ensuring a stable discharge plateau.

During the late discharge stage (Fig. 10c), a dynamic equilibrium is established between the exfoliation and deposition of discharge products, leading to the formation of a stable porous architecture. The continuous propagation of micro-crack networks renews the active reaction interfaces, while the presence of NdBi phase effectively suppresses localized excessive dissolution of the matrix, preventing the “chunk effect”. Furthermore, the timely escape of H_2_ bubbles mitigates electrode passivation, further enhancing reaction stability. Owing to these structural optimizations, the Mg-1Bi-0.5Nd alloy achieves a remarkably high discharge efficiency of 68.5% at a current density of 20 mA cm^−2^.

## Analysis

### Strategic control of second phases for mitigating galvanic corrosion

The distribution and composition of secondary phases critically determine the electrochemical stability of Mg-Bi-RE anodes. EDS analysis (Table 2) reveals that Mg-1Bi-0.5Nd forms a unique semi-continuous NdBi network (area fraction: 1.3%), while other alloys (e.g., Mg-1Bi-0.5Y) exhibit coarse blocky phases (e.g., MgYBi). This structural distinction yields two key advantages. The first aspect is the suppression of micro-galvanic corrosion suppression: the fine NdBi network homogenizes current distribution, reducing localized dissolution at phase boundaries. In contrast, isolated MgYBi/CeBi/LaBi phases in other alloys act as strong cathodes, accelerating α-Mg matrix dissolution (Fig. 8c vs. 8a, g,i). The superior performance of the semi-continuous NdBi network in this work stands in stark contrast to the detrimental effect of coarse, isolated cathodic phases reported in other systems. For example, in Mg-Y-based alloys, coarse MgYBi phases act as strong cathodic sites, severely accelerating the micro-galvanic corrosion of the α-Mg matrix and leading to rapid performance decay. Similarly, our own results for the Mg-1Bi-0.5Y alloy (with coarse MgYBi phases, Fig. 3c,d) confirm this drawback, showing the worst corrosion and discharge performance among the five alloys studied. The structure of the secondary phase is therefore more critical than its mere presence. The network-distributed NdBi phase in our alloy not only minimizes the cathode-to-anode area ratio but also strengthens the interface, effectively anchoring the discharge products and mitigating the chunk effect. This mechanistic insight provides a new design strategy beyond simply adding alloying elements, emphasizing the control of phase morphology. The second aspect is “Chunk Effect” mitigation: The cohesive NdBi network anchors discharge products, preventing massive detachment of unreacted metal (validated by 45% lower weight loss vs. Mg-1Bi-0.5Y). This explains the 68.5% anodic efficiency of Mg-1Bi-0.5Nd at 20 mA cm^−2^ (Table 6).

### Grain refinement synergy: corrosion resistance & reaction kinetics

Grain size directly governs both corrosion behavior and discharge dynamics. The first aspect is corrosion barrier enhancement: Mg-1Bi-0.5Nd’s ultra-fine grains (557 μm, Fig. 1c) provide high-density grain boundaries that facilitate rapid formation of a protective a dense Mg(OH)_2_ layer. EIS data confirms this: its charge transfer resistance (R_ct_= 57.35 Ω cm^2^, Table 4) is approximately 97 times higher than that of Mg-1Bi-0.5Y (0.59 Ω cm^2^), impeding electron transfer at the interface. The second aspect is reaction kinetics acceleration: Fine grains increase active sites for Mg dissolution, enabling stable high-current discharge (1492.54 mAh g^−1^ at 20 mA cm^−2^, Table 5). Coarse-grained alloys (e.g., Mg-1Bi-0.5Y, 1310 μm) suffer from sluggish ion diffusion, causing voltage fluctuations (Fig. 6d).

### Self-optimizing discharge product morphology

The loose, cracked a dense Mg(OH)_2_ layer on Mg-1Bi-0.5Nd (Fig. 9c) is pivotal for sustained discharge, functioning via. The first aspect is electrolyte accessibility: Microcracks (2–5 μm width, > 10 μm depth) enable continuous electrolyte penetration, maintaining reaction activity. The second aspect is autonomous exfoliation: Hydrogen bubbles accumulate in pores, generating internal pressure that spalls off passivating layers (Fig. 10). This is quantified by the highest low-frequency inductance (L = 452.2 H, Table 4), indicating efficient product detachment. A dense and poorly permeable a dense Mg(OH)_2_ layer on Mg-1Bi-0.5Y (Fig. 9b) blocks active sites, triggering rapid voltage decay.

### Synergistic Bi-Nd effects on oxide film stability

The Bi-Nd synergy uniquely enhances surface film properties. The first aspect is Nd-induced densification: Nd³⁺ incorporates into a dense Mg(OH)_2_ layer, reducing defect density (evidenced by the broadest phase angle peak in Fig. 5b). The second aspect is Bi-mediated hydrogen suppression: The high hydrogen overpotential of Bi curbs the parasitic hydrogen evolution reaction (HER, Reaction 2), thereby decreasing self-corrosion (I_corr = 27.03 µA cm^−2^ vs. 216.60 µA cm^−2^ for Mg-1Bi-0.5Y, Table 3). This synergy minimizes “dead mass” loss, boosting usable capacity by 51.5% vs. commercial AZ31. The synergy between Bi and Nd observed in the Mg-1Bi-0.5Nd alloy offers a unique advantage. While the beneficial effect of Bi in increasing hydrogen overpotential is known, and the ability of RE elements like Gd and Y to refine grains is well-documented, the combination of Bi with Nd specifically promotes the formation of a thermally stable and electrochemically favorable NdBi phase. This is different from, for example, the Mg3Bi2 phase formed in binary Mg-Bi alloys or the MgxREy phases in other RE-containing alloys. This NdBi phase is key to achieving a combination of grain refinement, a homogeneous and protective surface film, and efficient discharge product exfoliation—a set of features not simultaneously achieved in AZ31, AP65, or other common anode materials^37^. Therefore, this study not only presents a high-performance alloy but also identifies the Mg-Bi-Nd system and the formation of the NdBi phase as a particularly promising direction for future magnesium anode development.”

**Fig. 14:**
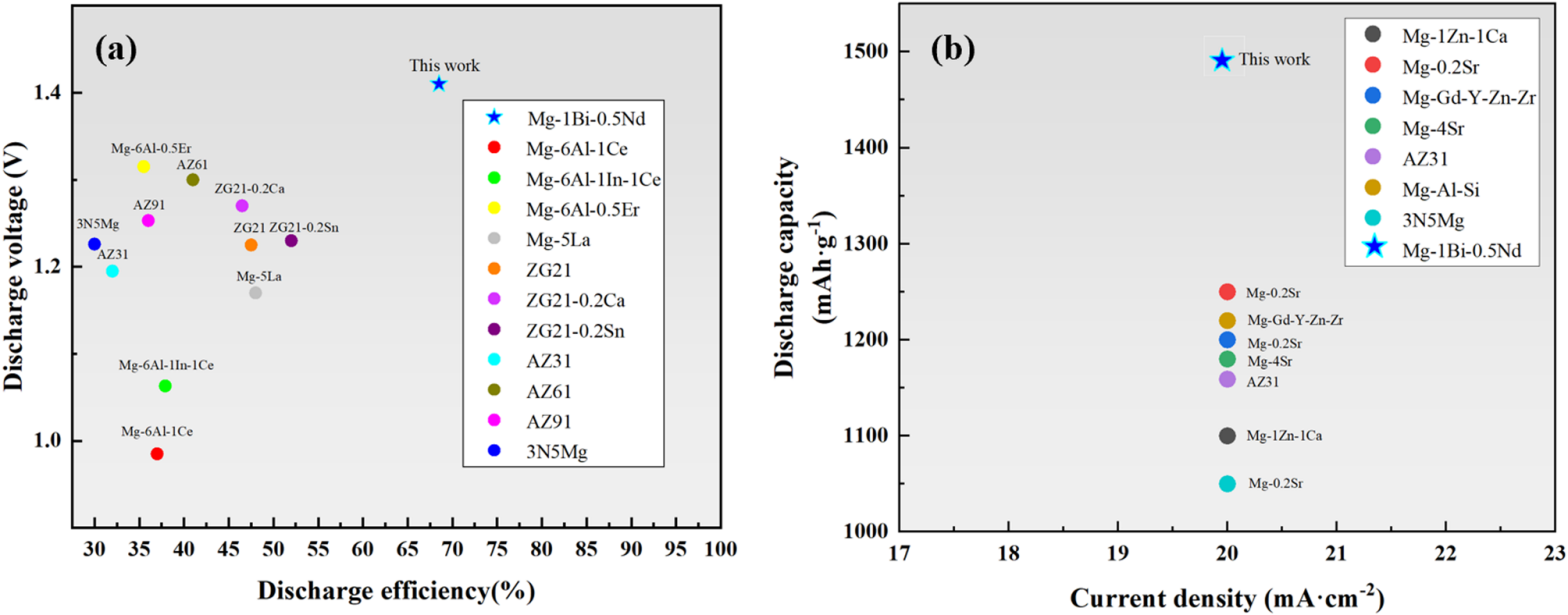
(**a**) Summary of magnesium alloy discharge efficiency; (**b**) summary of magnesium alloy discharge capacity.

Figure 14 is summary of magnesium alloy discharge efficiency and discharge capacity.

## Conclusions

This study systematically investigated the microstructure, electrochemical properties, and discharge performance of homogenized Mg-1Bi-0.5x (x = Gd, Y, Nd, Ce, La) alloys as anodes for magnesium-air batteries to explore high anodic efficiency. The main conclusions are as follows:

(1) The synergistic addition of rare earth elements and Bi significantly influenced the microstructure of Mg alloys. All five alloys exhibited uniform α-Mg matrix and secondary phase distribution. While the microstructural characteristics and phase composition were nearly identical among the five homogenized Mg alloys, the Mg-1Bi-0.5Nd alloy showed extensive precipitation of secondary phases along grain boundaries.

(2) Potentiodynamic polarization tests revealed that the Mg-1Bi-0.5Nd alloy possessed the highest charge transfer resistance (R_ct_), indicating the formation of a denser corrosion product film during corrosion that effectively impeded electron transfer and significantly improved corrosion resistance. Consequently, the Mg-1Bi-0.5Nd alloy demonstrated optimal corrosion resistance, whereas the Mg-1Bi-0.5Y alloy showed the poorest corrosion resistance.

(3) Discharge tests demonstrated that the Mg-1Bi-0.5Nd alloy exhibited the highest discharge voltage and most stable discharge curves across various current densities. At a discharge current density of 20 mA cm^−2^, it achieved a specific capacity of 1492.54 mAh g^−1^ and anodic efficiency of 68.5%, substantially outperforming other alloys. Surface morphology analysis indicated that the discharge products on the Mg-1Bi-0.5Nd anode exhibited a loose structure with cracks, which facilitated product detachment and enhanced the effective contact area between the anode and electrolyte, thereby improving discharge performance.

## Data Availability

The datasets generated and/or analyzed during the current study are available from the corresponding author on reasonable request.
